# The integration of artificial intelligence in assisted reproduction: a comprehensive review

**DOI:** 10.3389/frph.2025.1520919

**Published:** 2025-03-20

**Authors:** Pragati Kakkar, Shruti Gupta, Kasmiria Ioanna Paschopoulou, Ilias Paschopoulos, Ioannis Paschopoulos, Vassiliki Siafaka, Orestis Tsonis

**Affiliations:** ^1^ Assisted Conception Unit, Guy’s Hospital, Guy’s and St Thomas’ NHS Foundation Trust, London, United Kingdom; ^2^ Faculty of Medicine, School of Health Sciences, University of Ioannina, Ioannina, Greece; ^3^ School of Electrical and Computer Engineering, National Technical University of Athens, Athens, Greece; ^4^ School of Medicine, Faculty of Health Sciences, National and Kapodistrian University of Athens, Athens, Greece; ^5^ School of Health Sciences, University of Ioannina, Ioannina, Greece

**Keywords:** artificial intelligence, reproductive medicine, embryo selection algorithms, predictive modelling, IVF, ethics, personalised care, patient-centred

## Abstract

Artificial Intelligence (AI) has emerged as a transformative force in healthcare, with its integration into assisted reproduction technologies representing a notable milestone. The utilization of AI in assisted reproduction is rooted in the persistent challenge of optimizing outcomes. Despite years of progress, success rates in assisted reproductive techniques remain a concern. The current landscape of AI applications demonstrates significant potential to revolutionize various facets of assisted reproduction, including stimulation protocol optimization, embryo formation prediction, oocyte and sperm selection, and live birth prediction from embryos. AI's capacity for precise image-based analysis, leveraging convolutional neural networks, stands out as a promising avenue. Personalized treatment plans and enhanced diagnostic accuracy are central themes explored in this review. AI-driven healthcare products demonstrate the potential for real-time, adaptive health programs, fostering improved communication between patients and healthcare teams. Continuous learning systems to address challenges associated with biased training data and the time required for accurate decision-making capabilities to develop is imperative. Challenges and ethical considerations in AI-assisted conception as evident when taking into consideration issues such as the lack of legislation regulating AI in healthcare, a fact that emphasizes the need for transparency and equity in the development and implementation of AI technologies. The regulatory framework, both in the UK and globally, is making efforts to balance innovation with patient safety. This paper delves into the revolutionary impact of Artificial Intelligence (AI) in the realm of assisted reproduction technologies (ART). As AI continues to evolve, its application in the field of reproductive medicine holds great promise for improving success rates, personalized treatments, and overall efficiency. This comprehensive review explores the current state of AI in assisted reproduction, its potential benefits, challenges, and ethical considerations.

## Introduction

Artificial intelligence is “a technical and scientific field devoted to the engineered system that generates outputs such as content, forecasts, recommendations or decisions for a given set of human-defined objectives” (1) the way this works is by neural networks which are formed of nodal points that are interconnected and can relay information. This makes it capable of learning, reasoning, data and language processing, problem solving and perception. Machine learning and deep learning are concepts within AI which are distinct. Deep learning has been designed to have multiple layers of networks inspired by human brain. Most of these networks can do various tasks but are more suited to certain tasks like convolutional neural network for image analysis, generational adversarial network (creative AI) or recurrent neural network for language analysis (1).

Artificial Intelligence (AI) has traversed a remarkable trajectory, evolving from theoretical concepts to transformative tools that hold immense potential across various domains. The intersection of AI with healthcare has resulted in paradigm shifts, redefining how medical practices are conducted and revolutionizing patient outcomes. Within the realm of reproductive medicine, the integration of AI has introduced groundbreaking possibilities, reshaping the landscape of assisted conception. To comprehend this transformative journey, it is essential to delve into the historical development of AI, understand its applications in healthcare, and explore the rationale behind its integration into assisted reproduction (2). The inception of AI can be traced back to mid-20th century ideas, notably those of Alan Turing, who laid the theoretical groundwork for machines capable of intelligent behaviour. Despite early optimism, AI faced periodic setbacks, referred to as “AI winters,” marked by reduced funding and progress. Recent decades, however, have witnessed an unprecedented resurgence, fuelled by advances in computational power, the availability of vast datasets, and breakthroughs in machine learning algorithms (3, 4). This resurgence has propelled AI to the forefront of medical research, prompting exploration into its potential to address complex challenges within assisted reproduction (5).

Assisted reproduction technologies, including *in vitro* fertilization (IVF), have long grappled with challenges such as optimizing success rates, enhancing diagnostic precision, and navigating ethical considerations. The integration of AI into assisted conception processes heralds a new era, offering solutions to longstanding dilemmas. AI's capacity to swiftly and accurately analyse large datasets, coupled with its ability to identify intricate patterns, positions it as a game-changer in optimizing treatment plans, selecting viable embryos, and personalizing patient care. The augmentation of traditional reproductive methods with AI not only seeks to improve success rates but also addresses inefficiencies, streamlines decision-making, and introduces innovative approaches to diagnosis and treatment (6). Assisted reproduction technologies, encompassing procedures like IVF, intracytoplasmic sperm injection (ICSI), and preimplantation genetic testing (PGT), have transformed fertility treatment. These technologies aim to overcome various infertility challenges by facilitating conception outside the body and selecting embryos with the highest chances of successful implantation (7, 8).

The evolution of AI in healthcare mirrors its broader development. From early conceptualizations to contemporary breakthroughs, AI has progressively become an indispensable tool. In healthcare, AI applications range from diagnostic support and personalized treatment recommendations to administrative tasks, optimizing patient care, and revolutionizing medical research. The rationale behind integrating AI into assisted reproduction is multifaceted. AI's analytical prowess allows for rapid and precise processing of vast datasets, enabling personalized treatment plans based on individual patient characteristics (2). The ability to discern intricate patterns enhances diagnostic accuracy, contributing to the selection of viable embryos and optimizing overall success rates. AI's integration addresses longstanding challenges within assisted reproduction, fostering innovation and ushering in a new era of more efficient and personalized fertility treatments (9).

This narrative review aims to offer a panoramic exploration of the integration of AI in assisted reproduction. By synthesizing insights from diverse studies and perspectives, we seek to provide a nuanced understanding of AI's impact on personalized treatment plans, diagnostic accuracy, ethical considerations, and the future directions of this burgeoning field. Each section of this comprehensive review is penned by different authors, offering unique insights into specific facets of AI's role in assisted conception. Through an interdisciplinary lens, we aim to illuminate the promises, challenges, and ethical dimensions inherent in this evolving marriage of technology and reproductive medicine. Our endeavour is to equip practitioners, researchers, and stakeholders with a holistic understanding that can inform future advancements, foster ethical practices, and ultimately elevate the standard of care in assisted reproduction.

## Material and methods

For this review, four major search machines were explored MEDLINE, Google Scholar, PubMed and EMBASE, using the following keywords in combination: “Artificial Intelligence” AND “fertility” OR “assisted conception”. A broad search was decided by all authors to ensure all applications of Artificial Intelligence (AI) in the fertility sector can be identified and discussed. Data was collected regarding the role of AI in IVF, including sperm, oocyte, and embryo selection. Information regarding novel techniques and ethical implications has been also explored. All articles included were written in English. In selecting studies for inclusion in this review, a preference was given to those presenting original data rather than narrative reviews. Original studies contribute firsthand evidence and insights to the field, enhancing the robustness of the analysis. Notably, systematic reviews were considered in cases where a review format was deemed appropriate, providing a comprehensive synthesis of existing evidence. The decision to prioritize original data and include systematic reviews aligns with the aim of ensuring a rigorous and evidence-based approach to the synthesis of information presented in the main text.

## Current landscape of AI in assisted reproduction

Despite years of advancements in assisted reproductive techniques (ART), success rates remain suboptimal. The integration of new technologies, such as artificial intelligence (AI) and machine learning, holds promise for improving outcomes and introducing automation and standardization across clinics and countries (10–13). specific advancements in optimizing stimulation protocols, predicting embryo formation, and enhancing selection processes, highlight the potential of AI. Bringing automation and precision to image-based analysis aligns seamlessly with the goals of ART (14). Effective annotation can add speed, accuracy, and reproducibility, specifically tailored to detect abnormalities. Computer vision with Convolutional Neural Networks (CNN), a type of deep neural network, are instrumental in image analysis tasks, often combined with other AI models for comprehensive image annotation (15). Convolutional neural networks are a feed forward neural network for deep learning specially designed for image recognition.

### Sperm assessment and selection

The high workflow in *in vitro* fertilization (IVF) laboratories emphasizes the need for automation in sperm assessment. Computer-Assisted Sperm Analysis (CASA), as low-level algorithm has been around from the 1980's. CASA that incorporate newer machine learning algorithms promise to streamline analysis of sperm motility and viability and reduce human error (16). CNN are again the favourable deep learning methods employed for object tracking to assess motility in these systems. [Choi, Jw., Alkhoury, L., Urbano, L.F. *et al*. An assessment tool for computer-assisted semen analysis (CASA) algorithms. *Sci Rep* **12**, 16830 (2022). https://doi.org/10.1038/s41598-022-20943-9] Noteworthy advancements include handheld devices and smartphone analysis, automating sperm concentration and viability calculations, showcasing the potential of AI in improving efficiency and reducing subjectivity (17, 18). The researchers have reported a high degree of accuracy for these algorithms.

### Oocyte selection

Recognizing the pivotal role of oocytes in future embryos, researchers have studied various morphological features correlated with fertilization potential. Features like giant oocytes, giant polar bodies, increased perivitelline space, presence of vacuoles, smooth endoplasmic reticulum, increased granularity and thick zona pellucida have been linked to poor fertilization. AI models, such as Convolutional Neural Networks, demonstrate high accuracy (86%)in predicting fertilization potential and embryo development based on oocyte morphology (19). Furthermore, ultrasound patterns could potentially facilitate effective follicular tracking and trigger decision-making based on imaging patterns (20).

### Embryo selection

As single embryo transfer becomes standard practice, grading embryos at different stages is crucial. AI algorithms differentiate between normal and abnormal fertilization, offering a valuable tool in zygote assessment. Additionally computer vision through neural networks exhibit potential in predicting blastocyst formation and differentiating aneuploid from euploid embryos (21, 22). They have also been used as witnessing tools as their ability to identify each embryo with certainty (23).

### Blastocyst stage embryos

Blastocyst assessment using AI involves challenges such as determining the optimal time point for training algorithms and digitizing the alphanumeric grading system. AI models incorporating numerical scoring, quantitative assays in the machine learning networks show promise in accurately predicting blastocyst potential and aneuploidy. Diagnostic accuracy ranged from 60%–80% based on the dataset and sensitivity for predicting euploid embryos ranged from 75%–95% (24–27).

In all the studies there exists a lot of heterogeneity. Different AI models have been used in all the studies and keep evolving, which therefore limits the generalization of the results, another limitation is that the most of the AI models have been trained on less than a few 100 images. As earlier mentioned how an algorithm performs is determined by the real-world data used to train the algorithm. If the data for training is scant it can be unbalanced for example having negative images more than positive images can affect the accuracy of the system. The other issue is that not all the prediction models are for clinical pregnancy, because if the outcome that is being predicted is live birth there can be variables other than embryo quality i.e patient age, uterine factor that can influence the outcome. These are the key areas where standardization is needed in the work that is being done.

Future directions may involve integrating genomics, combining non-invasive preimplantation genetic testing with image analysis, and considering factors beyond image analysis and embryo quality, such as endometrial factors and patient characteristics (28). Table 1 comprises of all available AI applications in assisted reproduction.

**Table 1:** Applications of AI in assisted reproduction.

Sperm Assessment and Selection • Automated analysis of sperm using AI (16) • Computer-Aided Sperm Analysis (CASA) (17) Oocyte Selection • Neural models for analyzing oocytes (18) • Prediction models for fertilization potential (1) Embryo Grading • AI-powered differentiation of embryos (11) • Prediction of blastocyst potential (9) Personalized Treatment Plans • Individualized treatment plans based on patient data (31) • AI optimization of stimulation protocols (32)

## Personalized treatment plans and enhancing diagnostic accuracy

The intersection of precision medicine “(form of medicine that uses information about a person's genes, proteins, environment, and lifestyle to prevent, diagnose, or treat disease.)” (28) and artificial intelligence (AI) marks a significant advancement in personalized healthcare. The collaboration, exemplified by genome-informed prescribing, signifies a transformative stride wherein real-time recommendations are derived from machine-learning algorithms predicting medication needs based on genomic information (29). Sensitizing AI to individual variability is vital, and the thorough testing of AI-based healthcare products, like IBM's Watson, becomes imperative to address potential biases in training datasets. The reliance on continuous learning systems for decision support tools introduces challenges related to unbiased initial datasets and the time required for accurate decision-making capabilities to evolve (30).

In tandem, the integration of AI and machine learning in healthcare holds promise for disease diagnosis, treatment suggestions, and medical imaging interpretation. Despite the rapid progress in information technology, the adoption of electronic health records (EHR) has been a catalyst for healthcare transformation. The potential of AI in enhancing diagnostic accuracy is exemplified in studies utilizing clinical-genetic datasets, where genetic information enhances the prediction of ovarian stimulation outcomes in *in vitro* fertilization. However, challenges persist in the adoption of standardized data formats, acquiring well-labelled data, and navigating regulatory and sociocultural prerequisites. Machine learning algorithms, including logistic regression, support vector machines, decision trees, and random forests, contribute to improving the success rates of Assisted Reproductive Technology (ART) (31). These algorithms leverage various parameters and AI image analysis to enhance the assessment of infertility. Additionally, diagnostic tools powered by AI exhibit immense potential in healthcare by integrating patient data for quicker and more precise diagnoses. These tools, incorporating sophisticated algorithms, craft personalized treatment plans, considering medical history, genetics, and responses to previous treatments. This approach minimizes trial and error in medication selection and enables real-time monitoring and adjustment of treatment plans, ultimately enhancing overall patient care. Responsible incorporation of AI into IVF stimulation aims to elevate clinical care, improving accessibility to more effective and efficient fertility treatments (32, 33).

## Enhancing patient-centred care with artificial intelligence

Effective communication is paramount in achieving optimal patient outcomes and delivering high-quality healthcare. Artificial Intelligence (AI) in e-health communication opens avenues for enhancing the effectiveness of health promotion programs. The incorporation of human intelligence capacities in computing through AI enables the development of sophisticated e-health communication features. These include intuitive human-computer interfaces, congruent interaction responses, customized reminders, responsive monitors adapting to users' experiences and physical/psychological states and engaging relational agents (30). These applications support patients in their pursuit of a healthy life, fostering better understanding and communication with their healthcare team through smart, adaptive, and immediate health programs (34).

AI-powered robotics technologies, as highlighted in recent research, contribute to personalized medicine by manufacturing treatments tailored to individual patient features. The wide variations in genetics, biochemistry, physiology, and behaviour among humans underscore the necessity of personalized medicines. AI, as emphasized in current studies, analyses vast patient data through machine learning algorithms to customize medical treatments based on individual genomes, lifestyles, and environmental circumstances. This approach aids healthcare professionals in following personalized treatments, choosing drugs more effectively, and reducing adverse reactions. Additionally, AI-powered virtual assistants play a crucial role in improving patient involvement and offering individualized support (32). The potential of AI, particularly Machine Learning (ML), in healthcare services is evident in its ability to predict the best and most individualized treatment for each patient. ML can analyse individual characteristics to enhance treatment outcomes. AI's impact extends to healthcare professionals, assisting them in tailoring therapies based on patients' genetic profiles and predicted responses, as well as monitoring and adjusting treatments in real-time. This personalized approach minimizes errors and reduces the risk of adverse drug reactions (35).

Furthermore, AI proves valuable in predicting semen quality by considering a spectrum of environmental, lifestyle, hormonal, and genetic factors. By assessing these factors, AI can predict semen quality with good accuracy, aiding in decision-making processes to improve treatment outcomes in infertility. Despite the numerous benefits of AI in patient-centred care, certain limitations exist. Testing the utility of AI in healthcare is essential to identify potential algorithmic faults. Observing AI and big data-based healthcare products in randomized clinical trials is imperative for optimizing outcomes. Ethical considerations, including privacy and security of patient data, algorithm bias, and the need for transparent decision-making, pose challenges that require careful attention (36).

## Challenges and ethical considerations

The pervasive integration of AI in medical realms, particularly in reproductive medicine, evokes optimism, yet its early developmental stage complicates the nuanced assessment of both risks and opportunities. Despite promising strides, few AI advancements have reached technical maturity or found widespread adoption in clinical practice, prompting a critical scrutiny of the ethical dimensions surrounding AI in healthcare. Recent reviews have underscored ethical considerations across four pivotal domains: research, patient autonomy, physician-patient relationships, and reproductive justice (37, 38). A significant challenge lies in the retrospective nature of many studies, with limited prospective randomized trials. Such studies pose a heightened risk of bias, deviate from established reporting standards, and frequently lack essential data and code availability. Addressing these challenges necessitates large-scale randomized controlled studies, essential for validating algorithms and advancing research in personalized diagnosis and treatment, medical expert systems, and AI-supported reproduction (39). A further challenge lies in the reliance on extensive and diverse datasets, with effective treatment models for specific cohorts not universally applicable to individuals, potentially causing conflicts between physicians and AI-generated recommendations. The obligation on physicians to adhere to AI-driven recommendations, even when lacking a complete understanding or agreement with the system's suggestions regarding diagnosis and therapy, raises concerns about patient safety and treatment outcomes. Additionally, questions of liability loom large as machine learning algorithms become more ubiquitous, particularly in cases where AI applications lead to treatment errors or incorrect diagnoses (39).

**Figure 1:**
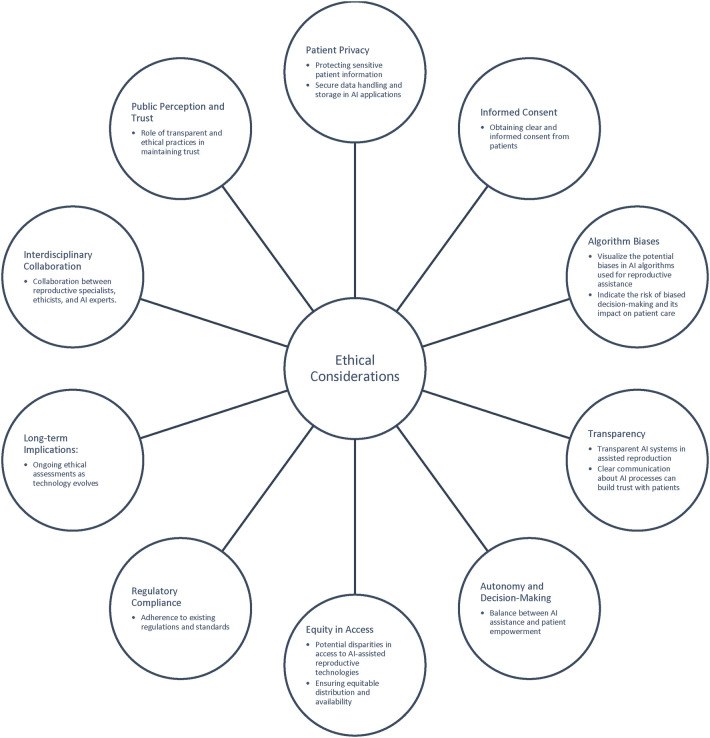
Ethical consideration of AI.

Meanwhile, ongoing AI research primarily concentrates on the image analysis of sperm cells and embryos, as well as predicting outcomes in assisted reproductive techniques. However, a substantial challenge emerges in effectively integrating AI into clinical practices due to the need for extensive and diverse datasets, coupled with variations in data definitions across clinics, patient demographics, and differences in clinical and laboratory guidelines. These challenges give rise to potential biases in AI tools, limiting their generalizability across diverse clinical settings. The “black box” nature of machine learning models, often lacking complete explainability, introduces complexity, as these partially transparent tools engage in automatic decision-making (40, 41). To navigate these ethical challenges and harness the full potential of transparent, patient-centred, and reliable AI-based algorithms for precision and improved outcomes, collaborative efforts between AI developers and healthcare professionals become imperative (42). This collaborative approach ensures a thoughtful examination of the quantity and quality of data used, emphasizing the necessity for transparency before the widespread implementation of AI in clinical practice (43). Figure 1 comprises of the main ethical concerns that arise from AI use in assisted reproduction.

## Future directions and innovations

The application of artificial intelligence (AI) in healthcare is poised to bring about significant transformations across various domains. AI's potential encompasses precise diagnoses, personalized treatment planning, early disease detection, predictive analyses, surgical enhancements through robotics, and remote patient monitoring (44). Moreover, its impact extends to optimizing healthcare operations, refining workflow processes, advancing precision imaging and radiology, streamlining drug discovery, and automating administrative tasks. The integration of AI into healthcare, however, poses ethical and regulatory challenges that warrant careful consideration (29, 31).

A distinct focus on personalized treatment strategies, drug prescriptions, and predictive analyses characterizes AI's role in the clinical workflow. Leveraging machine learning databases allows the assimilation of extensive patient data, enabling predictions based on individual medical histories and genetic profiles. This not only aids in anticipating disease outbreaks but also proves invaluable in epidemiological studies. Additionally, machine learning's rapid task completion and adept handling of vast datasets enhance diagnosis, treatment, and prognosis, with potential future advancements in AI-driven prevention products. These innovations, rooted in a deeper understanding of biology, may introduce novel risk assessment tools, such as “polygenic risk scores”, for calculating individual disease risks. The collaborative synergy between precision medicine and AI further envisions personalized prescriptions based on genotyping, incorporation of environmental factors into healthcare, classification of patients for therapy based on clinical factors, integration of social determinants of health into clinical workflows, and real-time assisted monitoring for various healthcare scenarios (30, 45). In assisted reproduction, AI emerges as a catalyst for reducing interfering factors, enhancing decision-making processes, and ultimately streamlining treatment procedures. Despite these promising strides, the implementation of generative AI in healthcare demands careful consideration, balancing its potential benefits—data collection, document translation, and diagnostic support—with critical challenges related to biases, trust issues, and privacy concerns (32).

## Regulatory framework and standardization

Presently, there is no dedicated legislation governing the use of artificial intelligence (AI) in healthcare. In the United Kingdom, AI in healthcare falls under existing laws, such as the UK Medical Device Regulations 2002 and the Data Protection Act 2018. The UK government has embraced a pro-innovation stance, aiming to foster technological growth while safeguarding patient interests and safety. The Medicines and Healthcare products Regulatory Agency (MHRA) designates AI as a Medical Device (AIaMD), treating it as a subcategory of Software as a Medical Device (SaMD). A comprehensive report published in November 2022 outlines the regulatory objectives, emphasizing pre-marketing and post-marketing analyses to ensure safety and fairness. The report underscores the need for technology to demonstrate generalizability and avoid introducing injustice or inequality. Key stakeholders, including the National Health Service (NHS), MHRA, and Care Quality Commission (CQC), are identified, encouraging public engagement to develop transparent guidelines that build trust in AI systems (46).

The World Health Organization (WHO) released regulatory considerations for AI in healthcare on October 19, 2023, providing guiding principles for its development. Meanwhile, the European Union (EU) proposed the AI Act in 2021, scheduled to take effect in late 2024. This legislation categorizes AI systems based on risk levels, ranging from minimal or no risk to limited risk, high risk, and unacceptable risk. The level of risk determines the applicability and market entry conditions for the technology. AI systems deemed to have unacceptable risk, posing threats to safety, rights, and livelihood, will be banned. High-risk systems can enter the market only if robust risk management systems are in place, complying with data regulations and accompanied by proper documentation (47).

Artificial intelligence (AI) is reshaping healthcare by facilitating disease diagnosis, treatment development, and enhancing patient care. However, the swift integration of AI in healthcare introduces ethical and legal challenges that necessitate effective regulation. Two primary approaches are considered: general regulation, applying broad rules across industries, and precise regulation, tailored to the unique considerations of healthcare AI. While some advocate for general regulation, others argue for the imperative of precise regulation in healthcare AI to ensure ethical and responsible usage. In the current regulatory landscape, the increasing prevalence of AI prompts calls for stricter regulations worldwide. Initiatives like the European Union's proposed AI Act aim to introduce a risk-based, ethical AI approach. Specialized regulations in healthcare, addressing issues like patient safety, data privacy, and medical liability are in need (48).

## Conclusion

In conclusion, the integration of Artificial Intelligence in assisted reproduction marks a paradigm shift in fertility treatments. AI offers unprecedented opportunities for personalized treatment plans, enhanced diagnostic accuracy, and patient-centred care. However, ethical considerations, regulatory frameworks, and the need for rigorous testing pose challenges that demand careful navigation. As AI continues to evolve, its potential to revolutionize assisted reproductive techniques is evident, paving the way for a future where precision and individualized care redefine the landscape of fertility treatments.
